# A Molecularly Imprinted Electrochemical Sensor Based on TiO_2_@Ti_3_C_2_T_x_ for Highly Sensitive and Selective Detection of Chlortetracycline

**DOI:** 10.3390/molecules28227475

**Published:** 2023-11-08

**Authors:** Linbo Deng, Jiawei Liu, Haiyan Huang, Changxi Deng, Limin Lu, Linyu Wang, Xiaoqiang Wang

**Affiliations:** Key Laboratory of Crop Physiology, Ecology and Genetic Breeding, Ministry of Education, College of Chemistry and Materials, Jiangxi Agricultural University, Nanchang 330045, China; a845831015@163.com (L.D.); 15180018556@163.com (J.L.); 13767967571@163.com (H.H.); dcxi80@sina.com (C.D.); lulimin816@126.com (L.L.)

**Keywords:** molecularly imprinted electrochemical sensor, Ti_3_C_2_T_x_, TiO_2_, polypyrrole, chlortetracycline

## Abstract

In view of the serious side effects of chlortetracycline (CTC) on the human body, it is particularly important to develop rapid, sensitive, and selective technologies for the detection of CTC in food. In this work, a molecularly imprinted electrochemical sensor with [Fe(CN)_6_]^3−/4−^ as signal probe was proposed for the highly sensitive and selective detection of CTC. For this purpose, TiO_2_, which acts as an interlayer scaffold, was uniformly grown on the surface of Ti_3_C_2_T_x_ sheets through a simple two-step calcination process using Ti_3_C_2_T_x_ as the precursor to effectively avoid the stacking of Ti_3_C_2_T_x_ layers due to hydrogen bonding and van der Waals forces. This endowed TiO_2_@Ti_3_C_2_T_x_ with large specific surface, abundant functional sites, and rapid mass transfer. Then, polypyrrole molecularly imprinted polymers (MIPs) with outstanding electrical conductivity were modified on the surface of TiO_2_@Ti_3_C_2_T_x_ via simple electro-polymerization, where the pyrrole was employed as a polymeric monomer and the CTC provided a source of template molecules. This will not only provide specific recognition sites for CTC, but also facilitate electron transport on the electrode surface. The synergistic effects between TiO_2_@Ti_3_C_2_T_x_ and polypyrrole MIPs afforded the TiO_2_@Ti_3_C_2_T_x_/MIP-based electrochemical sensor excellent detection properties toward CTC, including ultra-low limits of detection (LOD) (0.027 nM), a wide linear range (0.06–1000 nM), and outstanding stability, reproducibility, selectivity, and feasibility in real samples. The results indicate that this strategy is feasible and will broaden the horizon for highly sensitive and selective detection of CTC.

## 1. Introduction

Chlortetracycline (CTC) is a broad-spectrum antibiotic that has been frequently used in the past for disease prevention and treatment for humans and animals due to its efficiency against various types of Gram-positive and Gram-negative bacteria [1]. However, research has found that hard degradable CTC will inevitably lead to residues in animal-derived foods, which accumulate in the human body through the food chain. These residues will cause gastrointestinal disorders, liver and kidney damage, allergic reactions, antibiotic resistant infections, and other threats to human health [1,2,3]. Based on this, it is of great significance to develop highly sensitive and selective detection technology for the monitoring of trace CTC in food. So far, a series of technologies such as high-performance liquid chromatography (HPLC) [4], Raman spectroscopy [5], immunoassays [6], high-performance liquid chromatography–mass spectrometry (HPLC-MS) [7], fluorescence [8,9], and capillary electrophoresis [10] have been proposed to detect CTC. However, expensive apparatuses (HPLC, MS, etc.), complex operating procedures (cleaning the chromatographic columns, etc.), and time-consuming pretreatments (sample pretreatment and bottling, etc.) make simple and rapid detection of CTC a great challenge. Therefore, electrochemical methods have attracted considerable attention by virtue of their easy operation, high sensitivity, low cost, and easy miniaturization [11,12]. Despite the rapid development of electrochemical sensors in recent years, there are still some inherent drawbacks in their application, such as poor selectivity due to the presence of interference between many matrixes (amino acids, dopamine, uric acid, ascorbic acid, etc.) in biological tissues. Only some enzymes or antigens can achieve selective detection, but their high cost, complex preparation, and denaturation under adverse environmental conditions (such as pH, temperature, and chemicals) lead to a short lifetime of the sensors, limiting their practical applications [13,14,15]. Thus, the development of new strategies to improve the selectivity of electrochemical sensing techniques is imminent.

Molecularly imprinted polymers (MIPs) stand out for their target-driven, predetermined molecular recognition ability in the construction of highly selective electrochemical sensors [16,17]. MIPs are synthesized through polymerization of functional monomers, cross-linkers, and template molecules. After removing the template molecules, three-dimensional cavities are generated within the polymer matrix that is complementary to the template molecules in shape and size, allowing for further recognizing and rebinding of the target analytes [18,19]. Among the various preparation methods for MIPs, electro-polymerization stands out in the construction of imprinted electrochemical sensors due to its unique advantages, such as a strong adherence to the electrode surface, easy and rapid preparation, and a high reproducibility. At the same time, the morphology, uniformity, and thickness of the formed polymer film can also be precisely regulated by varying the electrochemical parameters [19]. The selection of polymerizable functional monomers is a critical factor in the preparation of MIPs, which greatly affects the sensing performance of the imprinted sensors. Polypyrrole (PPy), as a representative conductive polymer, is widely utilized to construct MIP-based sensors for the specific detection of target molecules due to its high electrical conductivity, superior stability, proper redox properties, and good biocompatibility [20,21]. Furthermore, oxygen-containing groups on pyrrole (Py), such as carbonyl and hydroxyl groups, are introduced into polymeric backbone, which is beneficial for controlling the binding and removal of template molecules [22].

Since the substrate material exhibits a pivotal effect on the performance of imprinted electrochemical sensors, many functionalized nanomaterials with large specific surface areas and good conductivity have been developed for their construction [19]. For example, Chen et al. [23] synthesized ionic liquid functionalized MWCNT composite. The resulting MIP sensor showed remarkable analytical performance for CTC due to the excellent electrical conductivity and large specific surface area of MWCNT. Additionally, Liu et al. [24] fabricated an MIP sensor based on electrochemical reduction of graphene oxide. The introduced conductive graphene oxide improved the sensitivity of the sensor. MXene, a burgeoning-layered 2D transition metal carbide or carbonitride has received a lot of attention due to its remarkable conductivity, abundant functional groups, and hydrophilic layered surface [25]. However, the strong van der Waals force between adjacent layers causes severe self-stacking of MXene nanosheets, leading to the covering of some active sites and hindering substance diffusion between the layers and weakening the electrochemical sensing properties [26,27]. Introduced scaffolds, such as transitional metal chalcogenides (TMC) [28], transitional metal oxides (TMO) [29,30], polymers [31], and carbon materials [32], used to increase the spacing between MXene nanosheets have been demonstrated to be an effective approach to prevent MXene nonosheets from self-restacking. For example, Ma et al. inserted NH_2_-CNT into the interlayers of MXene via physical mixing to increase its interlayer spacing, effectively avoiding the self-restacking of MXene sheets [33]. Yang et al. used Ti_3_C_2_T_x_ MXene as the titanium source, and the TiO_2_ acted as a scaffold which was uniformly grown on the surface of the MXene sheet using a simple in situ growth method, which also effectively prevented the interlayer stacking of MXene [34]. In contrast, TiO_2_@MXene obtained via an in situ growth method using MXene as a precursor was more appealing, as avoided not only the instability of physical incorporation, but also the cumbersome synthesis process of chemical doping [35].

Herein, we developed a highly sensitive and selective molecularly imprinted polymer electrochemical sensor based on TiO_2_@Ti_3_C_2_T_x_ for the detection of CTC. Specifically, TiO_2_@Ti_3_C_2_T_x_ was synthesized via a simple two-step calcination using Ti_3_C_2_T_x_ as the precursor, and then the polypyrrole MIP was modified on the TiO_2_@Ti_3_C_2_T_x_/GCE surface through electro-polymerization with pyrrole as the polymeric monomer and CTC as the template molecule, followed by elution of CTC from the polypyrrole thin films. The uniform TiO_2_ on the Ti_3_C_2_T_x_ surface was employed as an interlayer spacer to effectively prevent the stacking of MXene sheets, endowing TiO_2_@Ti_3_C_2_T_x_ with fast ion transport abilities and a large specific surface area for MIP loading. Additionally, the three-dimensional cavities in polypyrrole MIPs enable selective recognition of CTC. After binding with CTC, the transport of electrons on the electrode surface was impeded, leading to a decrease in the signal current of [Fe(CN)_6_]^3−/4−^. Thus, [Fe(CN)_6_]^3−/4−^ was employed as a signal probe for detecting CTC. The synergistic effects of TiO_2_@Ti_3_C_2_T_x_ and MIPs conferred TiO_2_@Ti_3_C_2_T_x_/MIPs with a highly sensitive and selective detection performance for CTC. Additionally, the MIP/TiO_2_@Ti_3_C_2_T_x_-based electrochemical sensor demonstrated a similar reliability and recovery to that of HPLC for the detection of CTC in chicken and milk samples, proving the feasibility of the proposed sensor in the detection of real samples.

## 2. Results and Discussion

### 2.1. Characterization of the Materials

**Figure 1:**
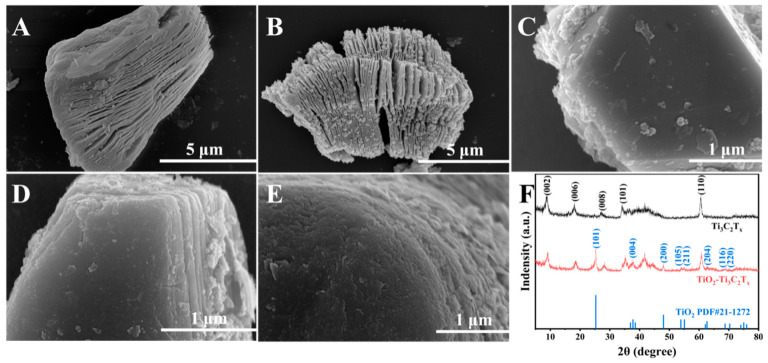
SEM images of Ti_3_C_2_T_x_ (**A**), TiO_2_@Ti_3_C_2_T_x_ (**B**), NIP/TiO_2_@Ti_3_C_2_T_x_/GCE (**C**), and MIP/TiO_2_@Ti_3_C_2_T_x_/GCE before (**D**) and after (**E**) treatment with elution. (**F**) XRD profiles of Ti_3_C_2_T_x_ (black) and TiO_2_@Ti_3_C_2_T_x_ (red).

The morphology structures of various materials were characterized using scanning electron microscopy (SEM). Figure 1A shows the typical accordion-like multilayer morphology of Ti_3_C_2_T_x_, indicating that the Ti_3_C_2_T_x_ was prepared successfully. After the two-step calcination, there were many particles on the surface and interlayer of the Ti_3_C_2_T_x_, and the interlayer spacing of the Ti_3_C_2_T_x_ was significantly larger (Figure 1B), demonstrating that the introduction of TiO_2_ nanoparticles as scaffolds was indeed effective in preventing the interlayer stacking of Ti_3_C_2_T_x_. Then, the morphologies of NIP/TiO_2_@Ti_3_C_2_T_x_ (Figure 1C) and MIP/TiO_2_@Ti_3_C_2_T_x_ before (Figure 1D) and after (Figure 1E) eluting were investigated. The results showed that there was no obvious difference between NIP/TiO_2_@Ti_3_C_2_T_x_ and MIP/TiO_2_@Ti_3_C_2_T_x_ before eluting the CTC molecules, suggesting that the presence of CTC would not affect the electro-polymerization of pyrrole. Figure 1E shows that the surface of MIP/TiO_2_@Ti_3_C_2_T_x_ after eluting the CTC molecules became rougher, which might have been caused by removing the CTC from the polypyrrole thin membrane, revealing that the CTC was successfully eluted. Subsequently, the crystal structures of the Ti_3_C_2_T_x_ and TiO_2_@Ti_3_C_2_T_x_ samples were studied using X-ray diffraction spectroscopy (XRD) (Figure 1F). There were some sharp diffraction peaks at 8.84°, 18.10°, 27.22°, 34.08°, and 60.59°, corresponding to the (002), (006), (008), (101), and (110) crystal planes of Ti_3_C_2_T_x_, respectively [36]. After the calcination treatment, several additional diffraction peaks were observed at 25.28°, 37.80°, 48.05°, 53.89°, 55.06°, and 62.69° in the XRD pattern of TiO_2_@Ti_3_C_2_T_x_, which were ascribed to the (101), (004), (200), (105), (211), and (204) facets of anatase TiO_2_ (PDF#21-1272), respectively [37,38]. These results showed that the TiO_2_ particles were successfully formed on the Ti_3_C_2_T_x_ surface after the calcination treatment, with the original structure of Ti_3_C_2_T_x_ retained.

### 2.2. Electrochemical Characterizations

**Figure 2:**
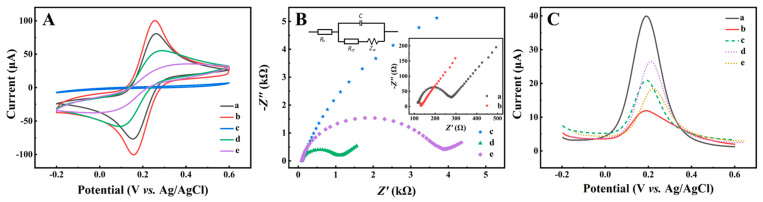
CV curves (**A**) and EIS (**B**) of bare GCE (curve a), TiO_2_@Ti_3_C_2_T_x_/GCE (curve b), MIP/TiO_2_@Ti_3_C_2_T_x_/GCE (before removal, curve c), MIP/TiO_2_@Ti_3_C_2_T_x_/GCE (after removal, curve d), and MIP/TiO_2_@Ti_3_C_2_T_x_/GCE (after rebinding, curve e) in 0.1 M KCl solutions containing 5 mM [Fe(CN)_6_]^3−/4−^. (**C**) DPV response of MIP/TiO_2_@Ti_3_C_2_T_x_/GCE (after removal, curve a), MIP/TiO_2_@Ti_3_C_2_T_x_/GCE (after rebinding, curve b), NIP/TiO_2_@Ti_3_C_2_T_x_/GCE (curve c), MIP/GCE (after removal, curve d), and MIP/GCE (after rebinding, curve e) in 0.1 M KCl solutions containing 5 mM [Fe(CN)_6_]^3−/4−^.

Cyclic voltammetry was used to electropolymerize the molecularly imprinted membranes in 0.1 M of phosphate-buffered solution (PBS, 0.1 M, pH = 6.0) containing 10 mM of Py and 1 mM of CTC (Figure S1). The electrochemical behaviors of the different electrodes were evaluated using cyclic voltammetry (CV) in a 0.1 M KCl solution containing 5 mM of [Fe(CN)_6_]^3−/4−^ (Figure 2A). A well-defined pair of redox peaks were observed on the CV curve of the bare glassy carbon electrode (GCE) (curve a), indicating that there was a characteristic reversible electron transfer process of [Fe(CN)_6_]^3−/4−^ on the electrode surface. After the modification of the TiO_2_@Ti_3_C_2_T_x_ composite, the redox peak current of the [Fe(CN)_6_]^3−/4−^ increased significantly (curve b). This phenomenon was attributed to the fact that TiO_2_@Ti_3_C_2_T_x_ significantly increased the electro-active surface area and conductivity of the electrode, thus effectively accelerating the electron transfer between the electrode and the redox probe. After the formation of MIP on the TiO_2_@Ti_3_C_2_T_x_/GCE surface before removing the CTC molecules, the peak current of [Fe(CN)_6_]^3−/4−^ disappeared almost completely (curve c). As expected, with the removal of the CTC molecules, a pair of obvious redox peaks was observed (curve d), demonstrating that the eluted polymer matrix became more permeable, which enabled a smoother diffusion of the redox probe. After binding with the CTC molecules, the current response of the [Fe(CN)_6_]^3−/4−^ significantly decreased, and the peak–peak potential difference increased (curve e), suggesting that the electron transport on the electrode surface was impeded. This could be interpreted as the cavities in the imprinted film being re-occupied by the template molecule, reducing the permeation efficiency of the film and blocking the penetration of the probe and electron. The results of the electrochemical impedance spectroscopy (EIS) (Figure 2B) were in perfect agreement with the CV results. The charge transfer resistance (R_ct_) of the TiO_2_@Ti_3_C_2_T_x_/GCE (130 Ω) was lower than that of the bare GCE (164.3 Ω), showing that the TiO_2_@Ti_3_C_2_T_x_ composite exhibited good conductivity. After being covered by the MIP films, the R_ct_ value of MIP/TiO_2_@Ti_3_C_2_T_x_/GCE was more than 5 kΩ, indicating that the MIP has been successfully modified. After the removal of the CTC molecules, the R_ct_ value of MIP/TiO_2_@Ti_3_C_2_T_x_/GCE was only 836.9 Ω, and its R_ct_ value increased to 3268 Ω after rebinding CTC molecules, demonstrating that the imprinted cavities within the MIP films were important channels for electron transfer on the modified electrode. All these results revealed that these electrodes were successfully prepared.

Since CTC is a non-electroactive substance (Figure S2), [Fe(CN)_6_]^3−/4−^ was chosen as the signal probe to achieve quantitative detection of CTC. Then, the DPV curves of various electrodes in a 0.1 M KCl solution containing 5 mM of [Fe(CN)_6_]^3−/4−^ were investigated to verify the feasibility of the strategy (Figure 2C). The results showed that after the removal of CTC, MIP/TiO_2_@Ti_3_C_2_T_x_/GCE exhibited the highest response current (curve a), while the response current of NIP/TiO_2_@Ti_3_C_2_T_x_/GCE (curve c) was much less. This phenomenon demonstrated that the abundant imprinted cavities in polypyrrole MIP could effectively promote electron transport on the electrode surface. After rebinding with CTC, a significant decrease in the peak current of [Fe(CN)_6_]^3−/4−^ was observed (curve b), indicating that the electron transfer on the electrode surface was severely impeded due to the full occupation of the imprinted cavities in the MIP. For comparison, the electrochemical behavior of [Fe(CN)_6_]^3−/4−^ on MIP/GCE was investigated. This showed that the peak current of MIP/GCE after removal of CTC (curve d) was almost half that of MIP/TiO_2_@Ti_3_C_2_T_x_/GCE after removal of CTC, revealing that the large surface area of TiO_2_@Ti_3_C_2_T_x_ facilitated the loading of MIPs, thus generating more cavities to promote electron transport. After rebinding with CTC, the peak current of [Fe(CN)_6_]^3−/4−^ on MIP/GCE slightly decreased (curve e), suggesting that the sensitivity of MIP/GCE was much worse than that of MIP/TiO_2_@Ti_3_C_2_T_x_/GCE. This demonstrated that the synergistic effects between the TiO_2_@Ti_3_C_2_T_x_ with a large surface area and the MIP with a specific recognition site made the use of MIP/TiO_2_@Ti_3_C_2_T_x_ for CTC detection feasible.

The electrochemical active surface area (EASA) of TiO_2_@Ti_3_C_2_T_x_/GCE was calculated to be 0.1551 cm^2^, as shown in Figure S3. Subsequently, CV experiments under different scan rates were carried out to research the electron transport properties of [Fe(CN)_6_]^3−/4−^ on TiO_2_@Ti_3_C_2_T_x_/GCE (Figure S4). The results revealed that the electron transfer coefficient (α) and electron transfer number (n) was 0.5 and 1, respectively. Furthermore, the electron transfer rate (k_s_) of [Fe(CN)_6_]^3−/4−^ on TiO_2_@Ti_3_C_2_T_x_/GCE was calculated to be 1.95 s^−1^, proving the rapid electron transfer process on the TiO_2_@Ti_3_C_2_T_x_/GCE surface. The calculation and detailed description of the electrochemical parameters of EASA, α, n, and k_s_ are all included in the Supplementary File.

### 2.3. Optimization of Experimental Parameters

**Figure 3:**
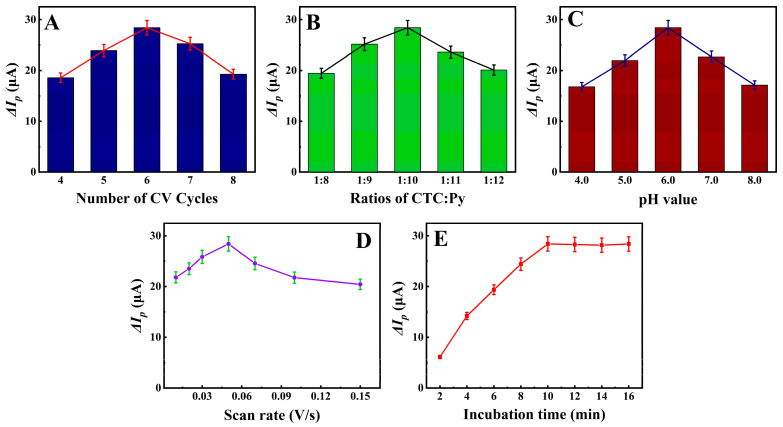
Optimization studies. (**A**) Cycle numbers of polymerization, (**B**) concentration ratio of template to monomer, (**C**) pH of the polymerization solution, (**D**) scan rate of polymerization, and (**E**) incubation time of the analyte. (All peak current responses to 0.4 μM of CTC were recorded using 5 mM [Fe(CN)_6_]^3−/4−^ as the redox probe in a 0.1 M KCl solution).

To obtain an optimal detection performance, several experimental conditions were investigated and optimized using differential pulse voltammetry (DPV) with [Fe(CN)_6_]^3−/4−^ as the electrochemical probe. The sensitivity and stability of the imprinted film are closely related to its thickness, which can be adjusted by controlling the cycle numbers of the electrochemical polymerization. Thus, the cycle numbers of CV were optimized, as shown in Figure 3A. The results showed that the difference between the peak current after elution and after rebinding of the template molecules (ΔI) increased significantly as the number of electro-polymerization cycles increased, until it started to decrease after six cycles. This may be explained by the fact that a film that is too thin does not provide enough imprinting sites and is prone to cracking. However, an undesired membrane thicknesses will make it difficult to remove the template molecules embedded deep within the polymer, resulting in poor site accessibility and a low binding capacity. The relationship between the thickness of the imprinted films and the number of CV polymerization cycles was investigated using atomic force microscopy (AFM) (Figure S5). As a result, six cycles were selected as the optimal number of cycles for electro-polymerization.

The molar ratio of the template molecule (CTC) to the functional monomer (pyrrole), which is related to the imprinting efficiency, was researched, and the results are shown in Figure 3B. It was found that the ΔI decreased gradually when the ratio was larger than 1:10. This phenomenon might be due to the possibility that excessive template molecules aggregated during electro-polymerization, resulting in the inability to form specific cavities. However, when the ratio was lower than 1:10, the binding cavities in the MIP films would decrease with the decrease in template molecules. Thus, the optimal molar ratio of template to monomer was 1:10.

Subsequently, the effects of the pH value of polymerization solution on the MIPs films were investigated (Figure 3C). It can be seen that the ΔI attained its maximum value at pH 6.0, which was chosen as the optimal pH value for further experiments.

The scan rate of electro-polymerization will affect the compactness of the polymer film, which in turn affects the available imprinting sites of the polymer film. Thus, the scan rate of the electro-polymerization was optimized, as shown in Figure 3D. It was observed that the ΔI increased rapidly with an increase in the scan rate, reaching a maximum at 50 mV/s. This result was explained by a compact and dense polymer film forming under a scan rate that was too slow and template molecules that were difficult to embed, thus reducing the available imprinting sites of the polymer film. As for the ΔI decreasing when the scan rate exceeded 50 mV/s, this should be attributed to a scan rate that was too fast, resulting in the formation of a rough and loose polymer film, which is not conducive to the stability and specificity of the polymer film. Therefore, 50 mV/s was chosen as the optimal scanning rate for the electro-polymerization.

Finally, the effects of the incubation time between the template molecules and binding sites on the sensing property were also examined. As shown in Figure 3E, the ΔI increased significantly at the initial stage and reached a plateau at 10 min, indicating that the prepared sensor possessed a rapid rebinding dynamic. The appropriate incubation time was thus chosen as 10 min for CTC in the subsequent measurements. The DPV plots of Figure 3 are in the Supplementary Files (Figure S6).

### 2.4. The Performance of MIP/TiO_2_@Ti_3_C_2_T_x_/GCE for Detecting CTC

**Figure 4:**
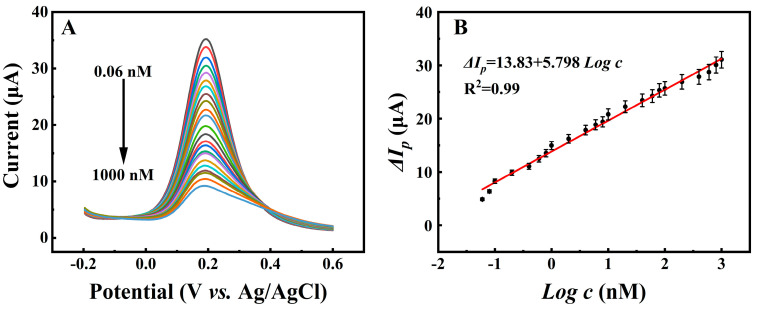
(**A**) DPV curves of incubated MIP/TiO_2_@Ti_3_C_2_T_x_/GCE in a 0.1 M KCl solution containing 5 mM of [Fe(CN)_6_]^3−/4−^ with CTC concentrations of 0.06, 0.08, 0.10, 0.20, 0.40, 0.60, 0.80, 1.0, 2.0, 4.0, 6.0, 8.0, 10, 20, 40, 60, 80, 100, 200, 400, 600, 800, and 1000 nM. (**B**) The corresponding linear calibration curve between the logarithm of the CTC concentration and ΔI_p_.

Under the optimal experimental conditions, the performance of the proposed MIP/TiO_2_@Ti_3_C_2_T_x_/GCE sensor for detecting CTC was explored. First, the MIP/TiO_2_@Ti_3_C_2_T_x_/GCE was incubated with different concentrations of CTC, and then the DPV curves of the MIP/TiO_2_@Ti_3_C_2_T_x_/GCE after incubation were investigated, as shown in Figure 4A. These results showed that the response current values decreased generally with an increase in the concentration of CTC, which was attributed to increasing numbers of CTC molecules being rebounded with the imprinted cavities in the MIP film, resulting in a blockage of electron transport on the electrode surface. In addition, there was a good linear relationship between the ΔI and the logarithm of the CTC concentration within the range of 0.06 nM to 1000 nM (R^2^ = 0.99). The standard calibration curve for the quantitative detection of the CTC is displayed in Figure 4B. According to this, the limit of detection (LOD) of the CTC was calculated to be 0.027 nM using the equation LOD = 3SD/m, where SD is the standard deviation of the blank (SD = 0.052) and m is the slope of the calibration plot (m = 5.798). The linear range was calculated to be 0.06–1000 nM, which was superior to the other reported electrochemical CTC sensors listed in Table 1 [23,24,39,40,41,42]. This confirmed the excellent sensing performance of the electrochemical CTC sensor based on MIP/TiO_2_@Ti_3_C_2_T_x_/GCE.

### 2.5. Reproducibility, Repeatability, Stability, and Selectivity Research

**Figure 5:**
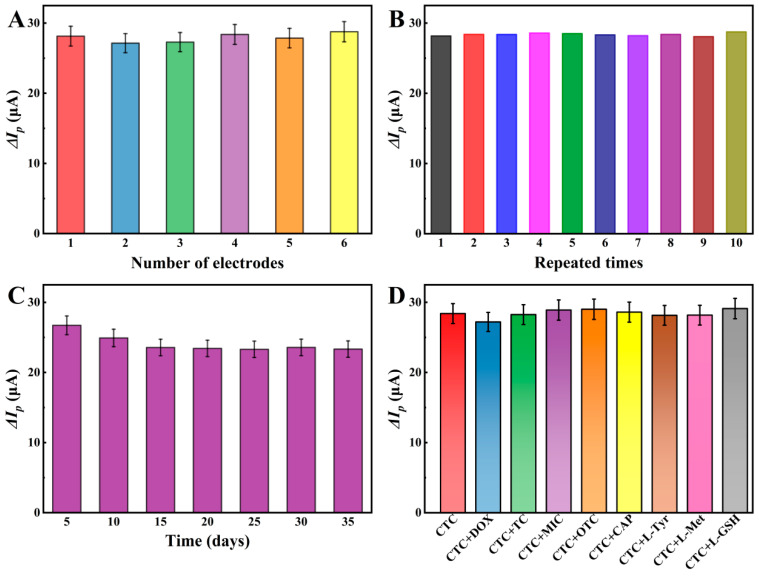
(**A**) Current response of six parallel MIP/TiO_2_@Ti_3_C_2_T_x_/GCEs to the same concentration CTC. (**B**) Reusability of the developed MIP/TiO_2_@Ti_3_C_2_T_x_/GCE sensor. (**C**) The stability of MIP/TiO_2_@Ti_3_C_2_T_x_/GCE. (**D**) Interference of other substances (20 μM) with CTC (0.4 μM).

The reproducibility of the proposed sensor was assessed by testing the ability of six parallel MIP/TiO_2_@Ti_3_C_2_T_x_/GCEs to detect 0.4 µM of CTC under the same conditions. Their relative standard deviation (RSD) value was calculated to be only 2.26% (Figure 5A), indicating that the as-fabricated MIP/TiO_2_@Ti_3_C_2_T_x_/GCE was highly reproducible for CTC sensing. The excellent repeatability of the MIP/TiO_2_@Ti_3_C_2_T_x_/GCE sensor was also proved by employing one electrode for ten repeated detections of CTC, with an RSD of 0.71% (Figure 5B). Furthermore, the long-term stability of MIP/TiO_2_@Ti_3_C_2_T_x_/GCE was evaluated based on its ability to detect CTC intermittently for five weeks using one electrode. This showed that the RSD of the seven detection results was only 5.1% (Figure 5C), revealing the satisfactory stability of MIP/TiO_2_@Ti_3_C_2_T_x_/GCE.

Finally, the selectivity of MIP/TiO_2_@Ti_3_C_2_T_x_/GCE for detecting CTC was investigated. Specifically, various potential interfering substances, whose concentrations were 50-fold higher than that of CTC, were sequentially added during incubation. The results indicated that all the substances, including the four CTC analogs (doxycycline, minocycline, tetracycline, and oxytetracycline), did not significantly interfere with the detection of CTC, demonstrating that the proposed sensor possessed excellent selectivity for CTC, even in the presence of common physiological interfering species. This phenomenon could be explained by the size, conformation, and the functional groups of the CTC molecule that matched well with the imprinted cavities in the MIP film. The DPV plots corresponding to Figure 5 are included the Supplementary files (Figure S7).

### 2.6. Real Sample Analysis

In order to verify the feasibility of the ability of MIP/TiO_2_@Ti_3_C_2_T_x_/GCE to detect CTC in real samples, it was utilized to detect CTC in chicken and milk. The results shown in Table 2 revealed that the recovery of CTC from real samples ranged from 97.9% to 102.4%, and the RSD was lower than 4%, indicating that the MIP/TiO_2_@Ti_3_C_2_T_x_/GCE was a promising candidate for the detection of CTC in real samples. Additionally, the detection results of MIP/TiO_2_@Ti_3_C_2_T_x_/GCE were close to that of HPLC, further demonstrating the feasibility of the sensor to detect CTC in real samples. Finally, MIP/TiO_2_@Ti_3_C_2_T_x_/GCE was used to detect CTC in chicken and milk without any treatment (Table S1), demonstrating that there was no CTC in the chicken or milk samples.

## 3. Experimental Section

### 3.1. Reagent

Ti_3_AlC_2_ (≥90 wt%) was purchased from Xianfeng Nanomaterials Technology Co., Ltd. (Nanjing, China). KH_2_PO_4_ (AR), K_2_HPO_4_ (AR), NaH_2_PO_4_ (AR), KCl (AR), and ethyl alcohol (AR) were obtained from Vita Chemical Reagent Co., Ltd. (Shanghai, China). K_3_[Fe(CN)_6_] (AR), K_4_[Fe(CN)_6_] (AR), HNO_3_ (AR), HClO_4_ (AR), HF (AR), N, N-dimethylformamide (DMF, AR), and acetonitrile (HPLC, ≥99.9%) were provided by Aladdin Biochemical Technology Co., Ltd. (Shanghai, China). Pyrrole (Py, AR), chlortetracycline hydrochloride (CTC, USP), doxycycline (DOX, ≥98%), chloramphenicol (CAP, ≥98%), L-methionine (L-Met, ≥99%), L-tyrosine (L-Tyr, ≥99%), and glutathione (GSH, ≥99%) were purchased from Yien Chemical Reagent Co., Ltd. (Shanghai, China). Minocycline hydrochloride (MIC, USP), tetracycline hydrochloride (TC, USP), and oxytetracycline hydrochloride (OTC, USP) were obtained from Sangong Bioengineering Co., Ltd. (Shanghai, China).

### 3.2. Apparatus

The electrochemical workstation (CHI760E, Shanghai, China) used in this study was equipped with a three-electrode system consisting of a working electrode, an Ag/AgCl electrode (reference electrode), and a platinum wire electrode (counter electrode). SEM images were obtained using a FEI Nova Nano SEM 450 scanning electron microscope (Brno, Czech Republic). XRD were collected using an X-ray diffractometer (Bruker D8 Advance, Mannheim, Germany). The HPLC analysis was carried out using a SHIMADZU LC-20AD HPLC (Kyoto, Japan) equipped with a SPD-40 UV detector.

### 3.3. Synthesis of TiO_2_@Ti_3_C_2_T_x_

The multilayered Ti_3_C_2_T_x_ was prepared using the HF etching method according to a previous report with minor modifications [43]. Briefly, 1 g of Ti_3_AlC_2_ powder was gradually added to 20 mL of HF solution (40%), and the mixture was stirred for 24 h at 40 °C. Then, the sediment was repeatedly washed with ultrapure water until the pH of the supernatant was 5–6. Finally, the Ti_3_C_2_T_X_ powder was collected through centrifugation and dried in a vacuum at 80 °C for 24 h.

The preparation of TiO_2_@Ti_3_C_2_T_x_ was carried out according to a previously reported approach with some modifications [35]. Firstly, 0.5 g of Ti_3_C_2_T_x_ was heated to 200 °C in a tube furnace under an air atmosphere for 1 h. Then, the powder was further calcined at 500 °C for 1 h in a tube furnace with a continuous nitrogen flow. The obtained black powder was labeled as TiO_2_@Ti_3_C_2_T_x_.

### 3.4. Preparation of TiO_2_@Ti_3_C_2_T_x_/GCE and MIP/TiO_2_@Ti_3_C_2_T_x_/GCE

Firstly, the bare GCE was polished with alumina powder and then sonicated with ultrapure water, absolute ethanol, and ultrapure water for 1 min in sequence. Then, 1 mg of TiO_2_@Ti_3_C_2_T_x_ was dispersed into 1 mL of ultrapure water and sonicated for 1 h to obtain a homogeneous suspension. Subsequently, 5 μL of the above suspension was dropped on the polished GCE surface and dried at room temperature to obtain TiO_2_@Ti_3_C_2_T_x_/GCE.

**Scheme 1:**
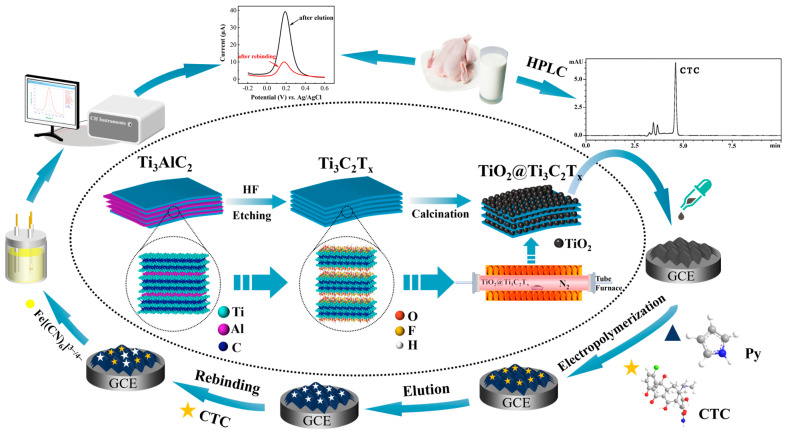
Schematic diagram of the preparation process of MIP/TiO_2_@Ti_3_C_2_T_x_/GCE and electrochemical detection of CTC.

Next, the obtained TiO_2_@Ti_3_C_2_T_x_/GCE was immersed in 10 mL of PBS (0.1 M, pH = 6.0) containing 10 mM of pyrrole and 1 mM of CTC, and the electro-polymerization process was carried out through CV scanning from −0.6 V to 1.0 V for 6 cycles using a scan rate of 50 mV/s. After the electro-polymerization, the CTC embedded in the polymerized film was eluted using a CV scanning range from −0.6 V to 1.2 V with a scan rate of 100 mV/s in PBS (pH = 7.0) until the current response was stable to obtain MIP/TiO_2_@Ti_3_C_2_T_x_/GCE. For comparison, the non-imprinted polymer (NIP)/TiO_2_@Ti_3_C_2_T_x_/GCE was also prepared according to the above-described procedure, except that no CTC molecules were added as template molecules. The preparation and detection process are shown in Scheme 1.

### 3.5. Electrochemical Measurements

Before the electrochemical measurement, the modified electrodes were incubated in PBS with a certain concentration of CTC for 10 min, followed by washing with ultrapure water. All of the electrochemical measurements were performed in a 0.1 M KCl solution containing 5.0 mM of [Fe(CN)_6_]^3−/4−^. The CV was carried out in a potential range from −0.2 V to 0.6 V with a scan rate of 50 mV/s, and the EIS was conducted in a frequency range between 100 kHz and 1 Hz, with initial potential of 0.2 V. The DPV responses were recorded from −0.2 V to 0.6 V, the potential incrementation was 4 mV, the pulse width was 0.05 V, the pulse amplitude was 0.05 V, and the pulse period was 0.5 s.

### 3.6. Preparation of Real Samples

The pretreatment process of the real samples referred to the national standard (GB/T 5009.116-2003, China) [44]. Milk and chicken samples were purchased from local markets, and the chicken was ground into mud. Then, 5.0 g of the samples (milk or homogenized chicken) were added into 25 mL of a 5% HClO_4_ solution, and ultrasound was applied for 30 min to extract their effective constituents. Finally, the supernatant was collected using centrifugation, filtered through a 0.22 μm filter membrane, and stored in refrigerator at 4 °C for further use.

## 4. Conclusions

In summary, we developed a MIP/TiO_2_@Ti_3_C_2_T_x_-based electrochemical sensor for the highly sensitive and selective detection of CTC, in which [Fe(CN)_6_]^3−/4−^ was employed as the signal probe. TiO_2_ was grown in situ on the surface of Ti_3_C_2_T_x_ as an interlayer spacer to hinder the interlayer stacking of Ti_3_C_2_T_x_, endowing TiO_2_@Ti_3_C_2_T_x_ with a large surface area and rapid mass transfer. Additionally, polypyrrole MIPs with outstanding electrical conductivity were covered on the TiO_2_@Ti_3_C_2_T_x_ surface via simple electro-polymerization. Due to the large surface area, rapid mass migration, fast electron transfer, and abundant specific recognition sites, the MIP/TiO_2_@Ti_3_C_2_T_x_-based electrochemical CTC sensor presented an ultra-low LOD (0.027 nM), wide linear range (0.06–1000 nM), and outstanding stability, reproducibility, and selectivity, even in the presence of some substances that are extremely similar to CTC. More importantly, the MIP/TiO_2_@Ti_3_C_2_T_x_/GCE displayed a remarkable ability to detect CTC in real samples. Thus, this work provides a new reference for sensitive and accurate detection of CTC using a simple strategy.

## Figures and Tables

**Table 1:** Comparison of the performance of MIP/TiO_2_@Ti_3_C_2_T_x_/GCE with other reported sensors for CTC detection.

Electrode	Detection Technology	Linear Range (μM)	LOD (μM)	References
IL1-SMIP/MWCNT-IL/GCE	LSV	0.4–55	0.08	[23]
BMMIPs/GCE	DPV	0.052–1044	0.052	[39]
RGO-MIP/GCE	DPV	10–500	-	[24]
CD-MWCNTs/PAMAM-Au/MIPs/GE	i-t	0.09–50	0.0495	[40]
SPGE	CV	5–50	0.58	[41]
BDDE	CV	500–50,000	0.01	[42]
MIP/TiO_2_@Ti_3_C_2_T_x_/GCE	DPV	0.00006–1	0.000027	This work

**Table 2:** Detection of CTC in real samples using MIP/TiO_2_@Ti_3_C_2_T_x_/GCE.

Sample	Number	Spiked Concentration (μM)	Determined by Target Sensor (μM)	Determined by HPLC (μM)	Recovery (%)	RSD (%)
Chicken	1	0.00	ND	ND	-	-
	2	0.20	0.197	0.184	98.7	3.91
	3	0.40	0.396	0.364	99	1.72
	4	0.80	0.783	0.688	97.9	2.51
Milk	1	0.00	ND	ND	-	-
	2	0.20	0.205	0.188	102.4	3.22
	3	0.40	0.394	0.364	98.5	2.95
	4	0.80	0.803	0.656	100.4	3.84

## Data Availability

The data presented in this study are available in the article.

## Supplementary Materials

The following supporting information can be downloaded at: https://www.mdpi.com/article/10.3390/molecules28227475/s1, Figure S1: Cyclic voltammograms taken during the electropolymerization of polypyrrole; Figure S2: CVs of bare GCE and TiO_2_@Ti_3_C_2_T_x_/GCE in the absence and presence of 0.4 μM CTC at pH 7.0 (0.1 M PBS); Figure S3: (A) CV plots of TiO_2_@Ti_3_C_2_T_x_ in 0.1 M KCl containing 5.0 mM [Fe(CN)_6_]^3−/4−^ at different scan rate (from 25 to 225 mV/s); (B) Linear relationships of peak current and square root of scan rate; Figure S4: (A) CV plots of TiO_2_@Ti_3_C_2_T_x_ in 0.1 M KCl containing 5.0 mM [Fe(CN)_6_]^3−/4−^ at different scan rate (from 25 to 225 mV/s); (B) Linear relationships of peak potential and logarithm of scan rate; Figure S5: AFM images of MIP films with different CV electro-polymerization cycles (A: 4 cycles, B: 6 cycles, C: 8 cycles), and the height profiles are inserted into the corresponding AFM images; Figure S6: DPV plots of optimization studies (A) cycle numbers of polymerization, (B) concentration ratio of template to monomer, (C) pH of polymerization solution, (D) scan rate of polymerization and (E) incubation time of analyte (The solid lines refer to the DPV responses before incubation, and the dashed lines refer to the DPV responses after incubation); Figure S7: DPV plots of (A) six parallel MIP/TiO_2_@Ti_3_C_2_T_x_/GCEs toward same concentration CTC, (B) 10 successive tests for the developed MIP/TiO_2_@Ti_3_C_2_T_x_/GCE sensor, (C) different days of MIP/TiO_2_@Ti_3_C_2_T_x_/GCE, and (D) different substances (20 μM) with CTC (0.4 μM); Table S1: Determination of CTC in real samples with MIP/TiO_2_@Ti_3_C_2_T_x_/GCE (The real samples were determined without any treatment).
