# Supplementary material for: A Molecularly Imprinted Electrochemical Sensor Based on TiO2@Ti3C2Tx for Highly Sensitive and Selective Detection of Chlortetracycline
Source: Molecules. 2023 Nov 8;28(22):7475. doi: 10.3390/molecules28227475 (PMC10673498; doi:10.3390/molecules28227475)
Supplement: Supplementary file 1 [file molecules-28-07475-s001.zip › molecules-2657327-supplementary.pdf]

Molecularly imprinted electrochemical sensor based on $TiO_2@Ti_3C_2T_x$ for highly sensitive and selective detection of chlortetracycline

Linbo Deng, Jiawei Liu, Haiyan Huang, Changxi Deng, Limin Lu, Linyu Wang* and Xiaoqiang Wang*

Key Laboratory of Crop Physiology, Ecology and Genetic Breeding, Ministry of Education, College of chemistry and materials, Jiangxi Agricultural University, Nanchang 330045, PR China

* Correspondence: linyuwang@jxau.edu.cn (L.W.); xqwang79@sina.com (X.W.)

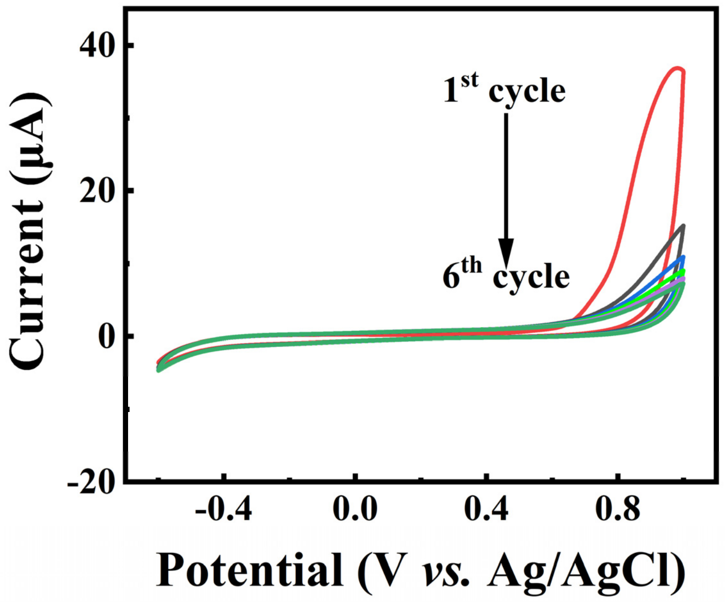

Figure S1. Cyclic voltammograms taken during the electropolymerization of polypyrrole.

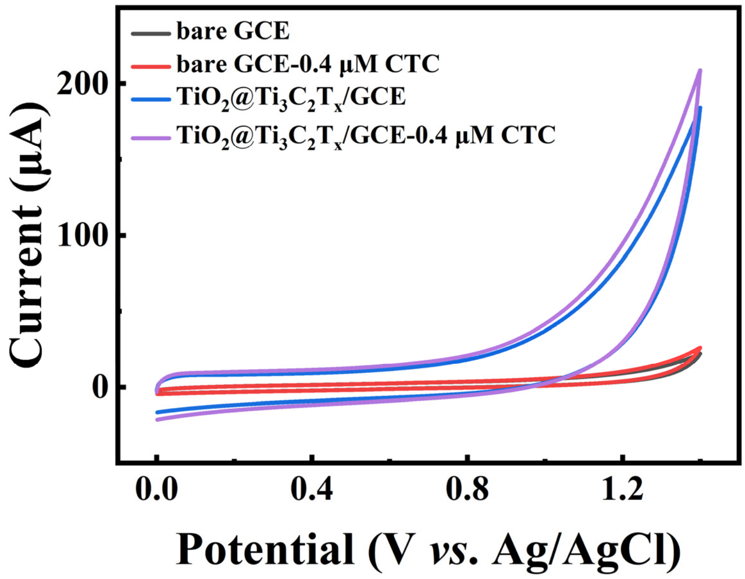

Figure S2. CVs of bare GCE and $TiO_2@Ti_3C_2T_x$/GCE in the absence and presence of 0.4 μM CTC at pH 7.0 (0.1 M PBS).

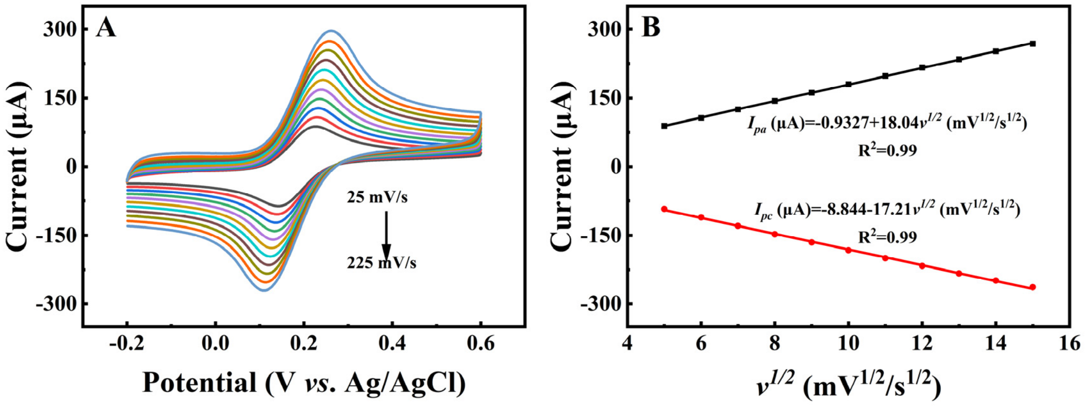

Figure S3. (A) CV plots of $TiO_2@Ti_3C_2T_x$ in 0.1 M KCl containing 5.0 mM $[Fe(CN)_6]^{3-/4-}$ at different scan rate (from 25 to 225 mV/s); (B) Linear relationships of peak current and square root of scan rate.

As shown in Figure S3A, the CV method was used to scan in 0.1 M KCl solution containing 5.0 mM $[Fe(CN)_6]^{3-/4-}$ at different scanning rates of 25 to 225 mV s^{-1}. The redox peak current could be obtained from Figure S3B (I_{pa} and I_{pc}) as follows:

$$I_{pa}\ (\mu A) = -0.9327 + 18.04\ v^{1/2}\ (mV^{1/2}/s^{1/2}),\ R^2 = 0.99 \qquad (S1)$$

$$I_{pc}\ (\mu A) = -8.844 - 17.21\ v^{1/2}\ (mV^{1/2}/s^{1/2}),\ R^2 = 0.99 \qquad (S2)$$

This indicated that the electrochemical sensing system followed a diffusion control mechanism. Calculate the electroactive surface area by the Randles-Sevcik equation:

$$I_p = 2.69 \times 10^5 n^{2/3} A D^{1/2} C v^{1/2} \qquad (S3)$$

where I_p (A) represents the peak current, n represents the number of electrons transferred in the $[Fe(CN)_6]^{3-/4-}$redox process, A (cm^2) is the electrical activity in the sensing system surface area, $[Fe(CN)_6]^{3-/4-}$ diffusion coefficient and concentration are represented by D (cm^2/s) and C (mol/cm^3), respectively, and v (V/s) is the scan rate. The results were as follows: the concentration of $[Fe(CN)_6]^{3-/4-}$ was 5.0 mM, n was 1, C was 5×10^{-6} mol/cm^3, and D was 6.7×10^{-6} cm^2/s. Therefore, based on the slope of I_p vs. $v^{1/2}$, the average electrochemical active surface area of $TiO_2@Ti_3C_2T_x$/GCE was calculated to be 0.1551 cm^2.

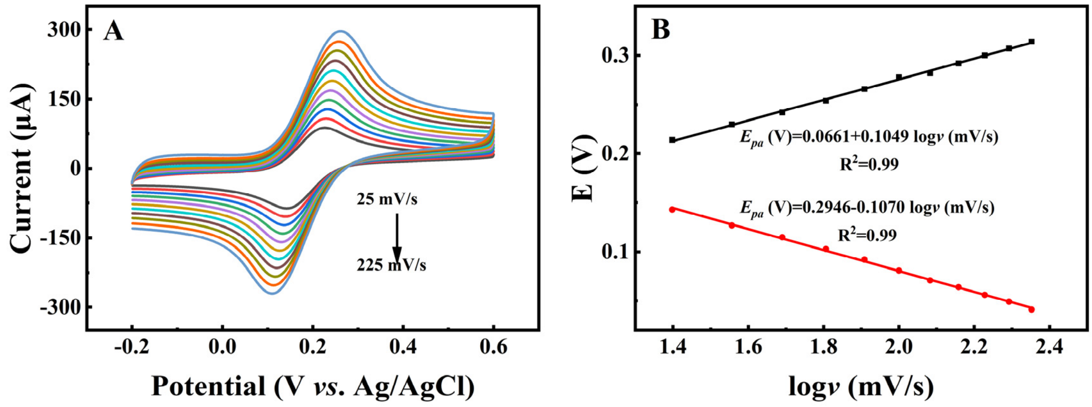

Figure S4. (A) CV plots of $TiO_2@Ti_3C_2T_x$ in 0.1 M KCl containing 5.0 mM $[Fe(CN)_6]^{3-/4-}$ at different scan rate (from 25 to 225 mV/s); (B) Linear relationships of peak potential and logarithm of scan rate.

Moreover, the anode peak potential (E_{pa}) and cathode peak potential (E_{pc}) had linear relationship with the logarithm of scan rate (log$v$) (Figure S4B). The linear regression equations were $E_{pa} = 0.0661 + 0.1049\log v$ ($R^2 = 0.99$) and $E_{pc} = 0.2946 - 0.1070\log v$ ($R^2 = 0.99$), respectively. According to the Laviron Eqs. (S4) and (S5), the slope of the equation for E_{pa} and E_{pc} could be represented as $2.3RT/n(1 - \alpha)F$ and $- 2.3RT/n\alpha F$, respectively.

$$E_{pa} = E^{0'} + [2.3RT/(1 - \alpha)nF]\log v \qquad \text{(S4)}$$

$$E_{pc} = E^{0'} - (2.3RT/\alpha nF)\log v \qquad \text{(S5)}$$

$$k_s = \alpha nFv/RT \qquad \text{(S6)}$$

Consequently, the electron transfer coefficient (α) and electron transfer number (n) could be calculated approximately as 0.5 and 1, respectively. Further, based on the Laviron Eqs. (S6), the electron transfer rate (k_s) of $TiO_2@Ti_3C_2T_x$/GCE were calculated to be 1.95 s^{-1}.

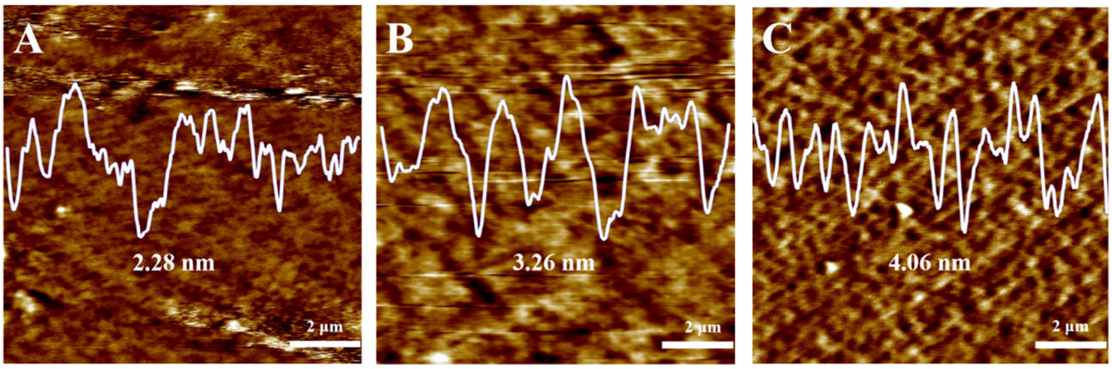

Figure S5. AFM images of MIP films with different CV electro-polymerization cycles (A: 4 cycles, B: 6 cycles, C: 8 cycles), and the height profiles are inserted into the corresponding AFM images.

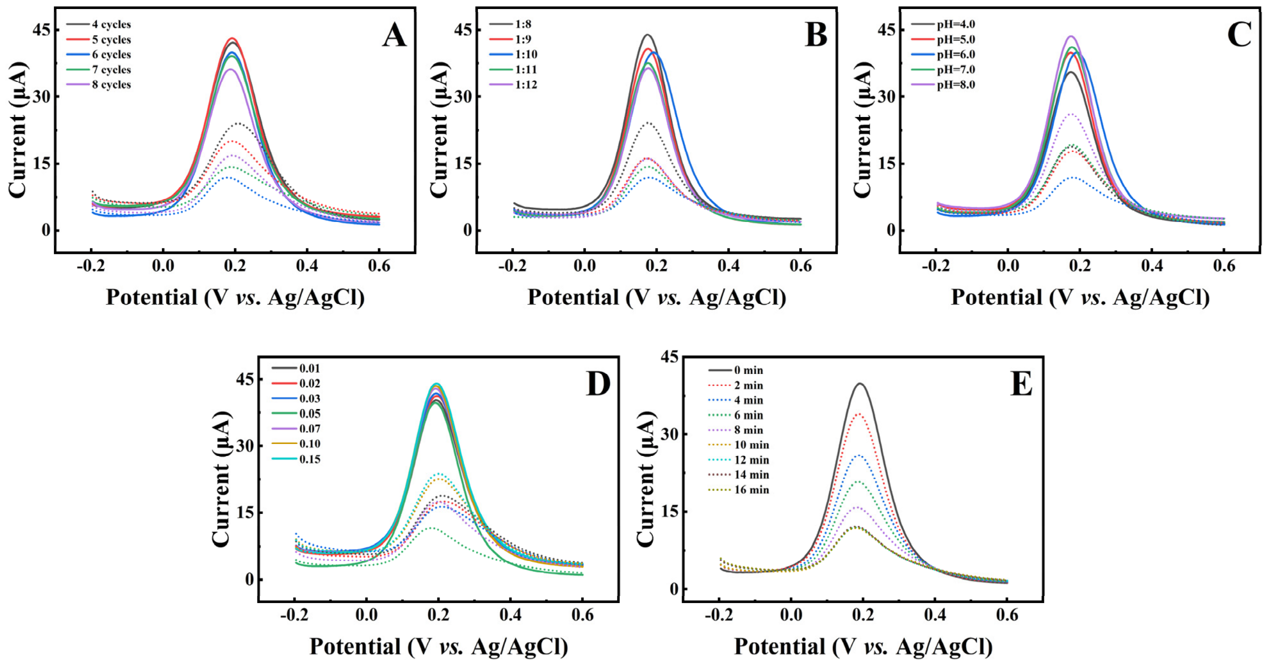

Figure S6. DPV plots of optimization studies (A) cycle numbers of polymerization, (B) concentration ratio of template to monomer, (C) pH of polymerization solution, (D) scan rate of polymerization and (E) incubation time of analyte. (The solid lines refer to the DPV responses before incubation, and the dashed lines refer to the DPV responses after incubation)

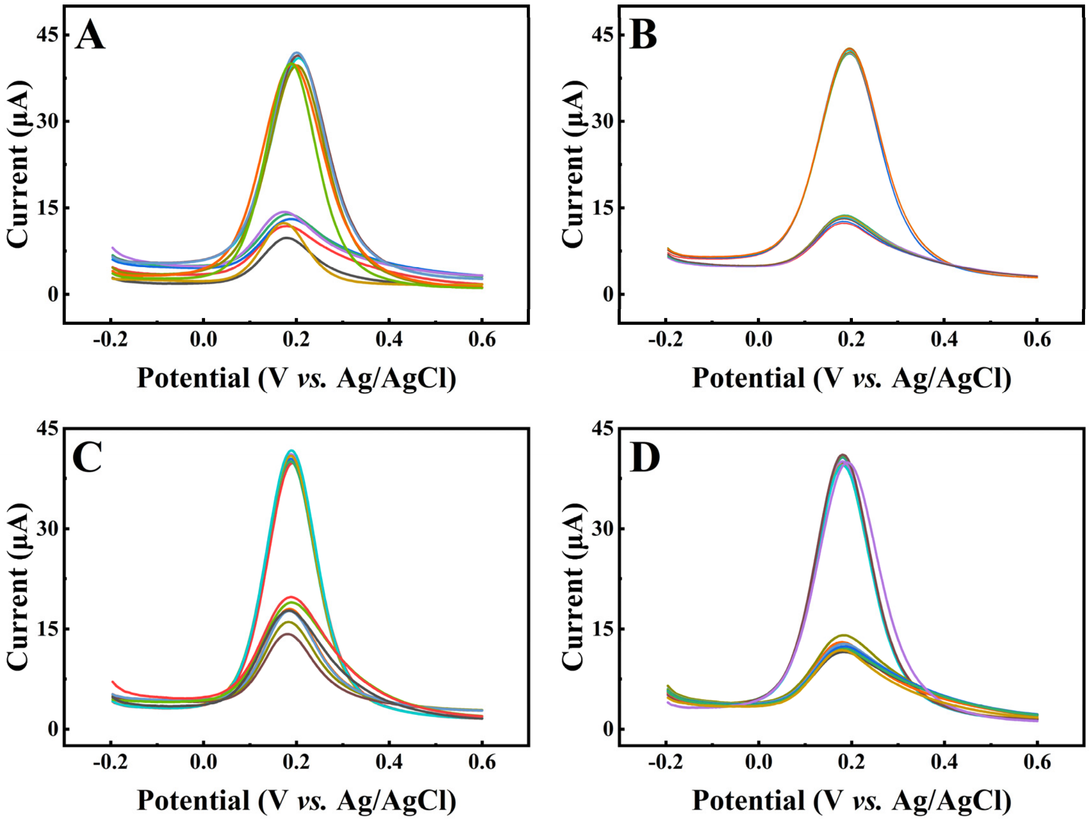

Figure S7. DPV plots of (A) six parallel MIP/TiO_2@$Ti_3C_2T_x$/GCEs toward same concentration CTC, (B) 10 successive tests for the developed MIP/TiO_2@$Ti_3C_2T_x$/GCE sensor, (C) different days of MIP/TiO_2@$Ti_3C_2T_x$/GCE, and (D) different substances (20 μM) with CTC (0.4 μM).

Table S1. Determination of CTC in real samples with MIP/TiO_2@$Ti_3C_2T_x$/GCE. (The real samples were determined without any treatment)

Sample	Number	Determined by target sensor (μM)	RSD (%)
Chicken	1	ND	-
	2	ND	-
	3	ND	-
Milk	1	ND	-
	2	ND	-
	3	ND	-
